# Comparative muscle development of scyphozoan jellyfish with simple and complex life cycles

**DOI:** 10.1186/s13227-015-0005-7

**Published:** 2015-04-17

**Authors:** Rebecca R Helm, Stefano Tiozzo, Martin K S Lilley, Fabien Lombard, Casey W Dunn

**Affiliations:** Brown University, 80 Waterman St. Box GW, Providence, 02912 RI USA; CNRS, Laboratoire de Biologie du Développement de Villefranche-sur-mer, Sorbonne Universités, UPMC Univ Paris 06, Observatoire Océanographique, 06230 Villefranche-sur-mer, France; Sorbonne Universités, UPMC Univ Paris 06, UMR 7093, LOV, Observatoire Océanologique, 06230 Villefranche-sur-mer, France; Current address: School of Biological and Chemical Sciences, Queen Mary University of London, Mile End Road, London, E1 4NS UK

**Keywords:** Scyphozoa, Pelagiidae, Adaptive decoupling hypothesis, Compartmentalization, Strobilation, Direct development

## Abstract

**Background:**

Simple life cycles arise from complex life cycles when one or more developmental stages are lost. This raises a fundamental question - how can an intermediate stage, such as a larva, be removed, and development still produce a normal adult? To address this question, we examined the development in several species of pelagiid jellyfish. Most members of Pelagiidae have a complex life cycle with a sessile polyp that gives rise to ephyrae (juvenile medusae); but one species within Pelagiidae, *Pelagia noctiluca*, spends its whole life in the water column, developing from a larva directly into an ephyra. In many complex life cycles, adult features develop from cell populations that remain quiescent in larvae, and this is known as life cycle compartmentalization and may facilitate the evolution of direct life cycles. A second type of metamorphic processes, known as remodeling, occurs when adult features are formed through modification of already differentiated larval structures. We examined muscle morphology to determine which of these alternatives may be present in Pelagiidae.

**Results:**

We first examined the structure and development of polyp and ephyra musculature in *Chrysaora quinquecirrha*, a close relative of *P. noctiluca* with a complex life cycle. Using phallotoxin staining and confocal microscopy, we verified that polyps have four to six cord muscles that persist in strobilae and discovered that cord muscles is physically separated from ephyra muscle. When cord muscle is removed from ephyra segments, normal ephyra muscle still develops. This suggests that polyp cord muscle is not necessary for ephyra muscle formation. We also found no evidence of polyp-like muscle in *P. noctiluca*. In both species, we discovered that ephyra muscle arises *de novo* in a similar manner, regardless of the life cycle.

**Conclusions:**

The separate origins of polyp and ephyra muscle in *C. quinquecirrha* and the absence of polyp-like muscle in *P. noctiluca* suggest that polyp muscle is not remodeled to form ephyra muscle in Pelagiidae. Life cycle stages in Scyphozoa may instead be compartmentalized. Because polyp muscle is not directly remodeled, this may have facilitated the loss of the polyp stage in the evolution of *P. noctiluca*.

**Electronic supplementary material:**

The online version of this article (doi:10.1186/s13227-015-0005-7) contains supplementary material, which is available to authorized users.

## Background

**Figure 1:**
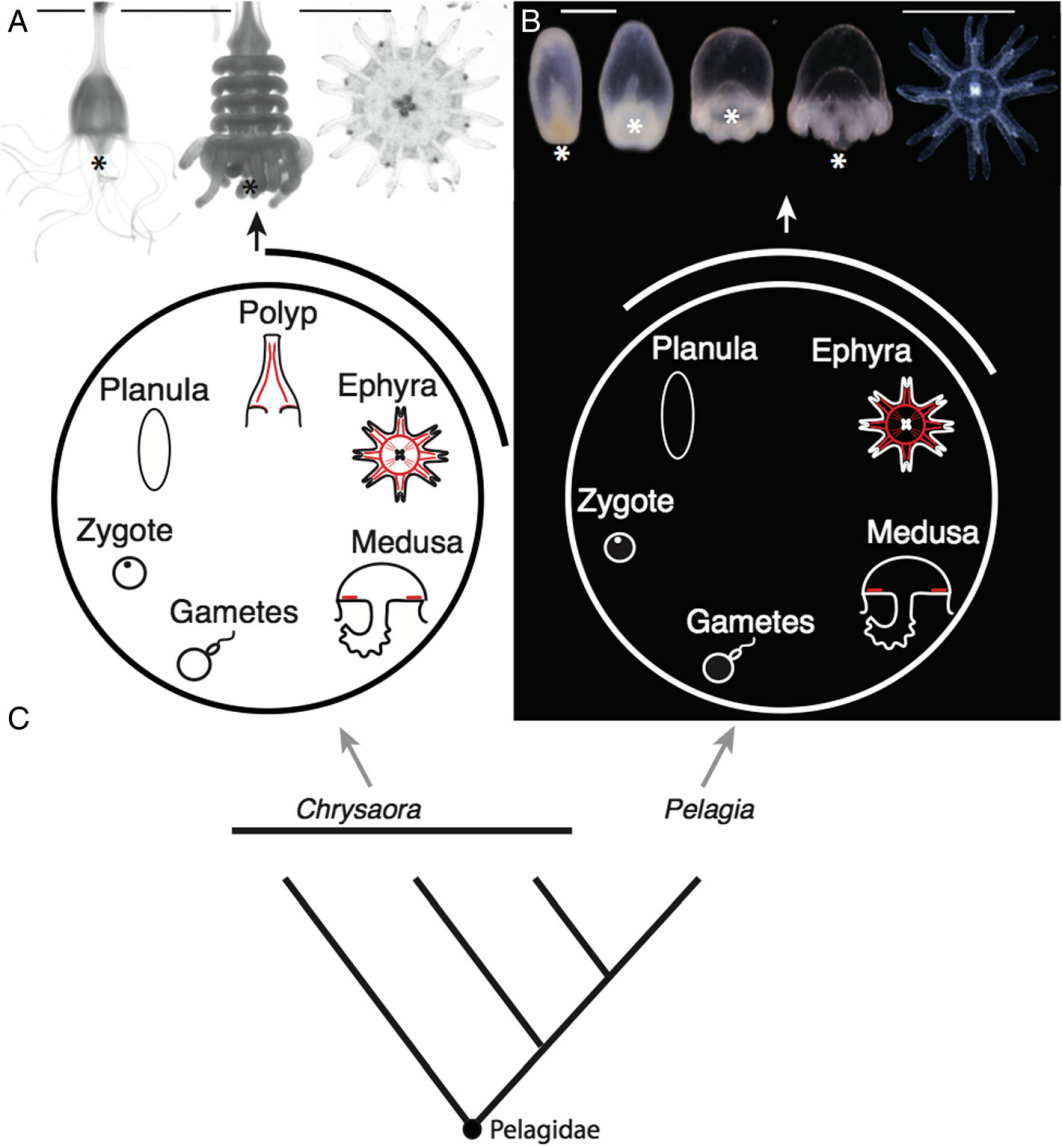
Scyphozoan life cycles. All animals are oriented oral down (the usual orientation for *Chrysaora quinquecirrha* polyps in nature) except ephyrae, which are oriented with the oral end facing the viewer. **(A)** Medusa development in the complex life cycle of *C. quinquecirrha* proceeds from a planula to a polyp with polyp cord muscle (red), to a strobila stage that liberates ephyrae, with circular muscle, radial muscle, and oral myoepithelial processes (red); picture scale bars are 1 mm. **(B)** Medusa development in *P. noctiluca*, by contrast, proceeds directly from a planula to an ephyra with circular muscle, radial muscle, and oral myoepithelial processes (red), left scale bar 300 μm, right scale bar (for ephyra only) 1 mm. In both species, ephyrae grow to medusae (with subumbrellar musculature (red)), which release eggs or sperm. **(C)** A simplified phylogeny of Pelagiidae, showing *P. noctiluca* nested within *Chrysaora*, based on [12]. * = mouth of polyps, ephyrae and developing *P. noctiluca* larvae.

The evolutionary transition between complex and simple life cycles has occurred many times [1-5], but how such a transition occurs presents a dilemma. If later developmental stages depend on earlier ones, how can a portion of development, such as a larval form, be abandoned, and development still proceed to a functional adult? With this question in mind, we set out to compare development between complex and simple life cycles in a group of jellyfish (Scyphozoa:Pelagiidae). There are currently 17 recognized species of Pelagiidae [6-8]. The medusae are all large, easily identifiable animals that have important impacts on ecosystems [9] and human health [10]. The canonical Pelagiidae life cycle begins when a fertilized egg develops into a ciliated non-feeding larva, termed a planula, which settles to the benthos and forms an asexually reproducing polyp (Figure 1A). Weeks to years later, this polyp fissions perpendicular to the oral-aboral axis in a process called strobilation, giving rise to multiple ephyrae (juvenile medusae), which grow into sexually reproductive adult medusae (Figure 1A). This complex life cycle is ancestral for Scyphozoa and Pelagiidae [11,12]. Within Pelagiidae, however, the mauve-stinger *Pelagia noctiluca* evolved a simpler life cycle [12-14]. Over several days, the ciliated larva of *P. noctiluca* transforms into a single small ephyra (Figure 1B), bypassing a benthic stage. This species spends its entire life in the water column. As the only species within Pelagiidae to have a simple life cycle (Figure 1C), *P. noctiluca* provides a unique opportunity to study the evolutionary simplification of a life cycle and its developmental implications.

Generally speaking, metamorphosis can be functionally classified into two types: compartmentalization and remodeling. Each has unique consequences for life cycle evolution [15]. Compartmentalization in life cycles can occur when adult features arise *de novo* from set-aside cell populations that remain quiescent in larvae (for example, imaginal disks in insects or the left rudiment in urchins). These set-aside cell populations can be composed of progenitor cells or multipotent cells destined to form adult tissues. In compartmentalization, loss of a larval stage may not strongly impact adult development because adult development is not directly dependant on larval morphology. In contrast, remodeling is the formation of adult features directly from larval structures [15], and thus larval structures may be necessary intermediates in development. For example, remodeling occurs in the insect nervous system; a subset of larval neurons withdraws their dendritic processes but do not die during metamorphosis. Instead, they are remodeled to form components of the adult nervous system [16]. In this way, adult structures depend on preexisting larval morphology.

To examine the potential role of compartmentalization and remodeling in life cycle evolution in Pelagiidae, we examined muscle morphology in *P. noctiluca* and a closely related species, *Chrysaora quinquecirrha*, which has a complex life cycle that includes a polyp. Each scyphozoan life cycle stage can be readily characterized by the presence or absence of unique muscle morphologies (Figure 1A,B illustrated red muscles), making muscle an excellent comparative character for understanding key aspects of development.

First, we examined polyp muscle morphology in *C. quinquecirrha*, to determine if polyp muscle is remodeled during strobilation and tested whether polyp muscle is necessary for ephyra muscle formation through experimental isolation of developing ephyra structures. Second, we characterized development of *P. noctiluca*, from embryo to ephyra, looking for evidence of polyp muscle. Third, we compared ephyra muscle development in both species, to discover how ephyra structures develop in these two radically different life cycles.

## Methods

### Animal collection and husbandry

Mature *Pelagia noctiluca* were collected off Villefranche-sur-mer, France, in August 2012 and June 2014. Medusae were housed at Observatoire Océanologique de Villefranche-sur-mer in a climate-controlled room kept at 18°C, on a 14-h light cycle. Four to five animals were housed per 20-l clear plastic bucket filled with 2-μm-filtered seawater; water was replaced twice daily, in the morning and evening. Animals were fed Golden Pearls (800 to 1,000 μm; brineshrimpdirect.com) once daily, and this was supplemented with live mixed plankton two to five times a week (as available). Medusae were maintained for several months. Spawning occurred roughly 2 to 3 h after light exposure, and eggs were collected immediately, stored in small glass dishes, and observed every few hours for evidence of fertilization. Development was asynchronous, and stages were collected based on visual identification.

*Chrysaora quinquecirrha* polyps were obtained from the New England Aquarium and maintained at Brown University in glass finger bowls. Polyps were kept at room temperature in the dark (to prevent excess algal growth). Animals were fed once weekly with newly hatched *Artemia* sp. (brineshrimpdirect.com), with water changed as needed. To induce strobilation, polyps were placed in a 50-μM indomethacin/seawater solution [17]. A subset of *C. quinquecirrha* ephyrae strobilated via this method were grown to sexual maturity, confirming healthy development from chemically induced strobilation.

### Fixation, phallotoxin staining and imaging

**Figure 2:**
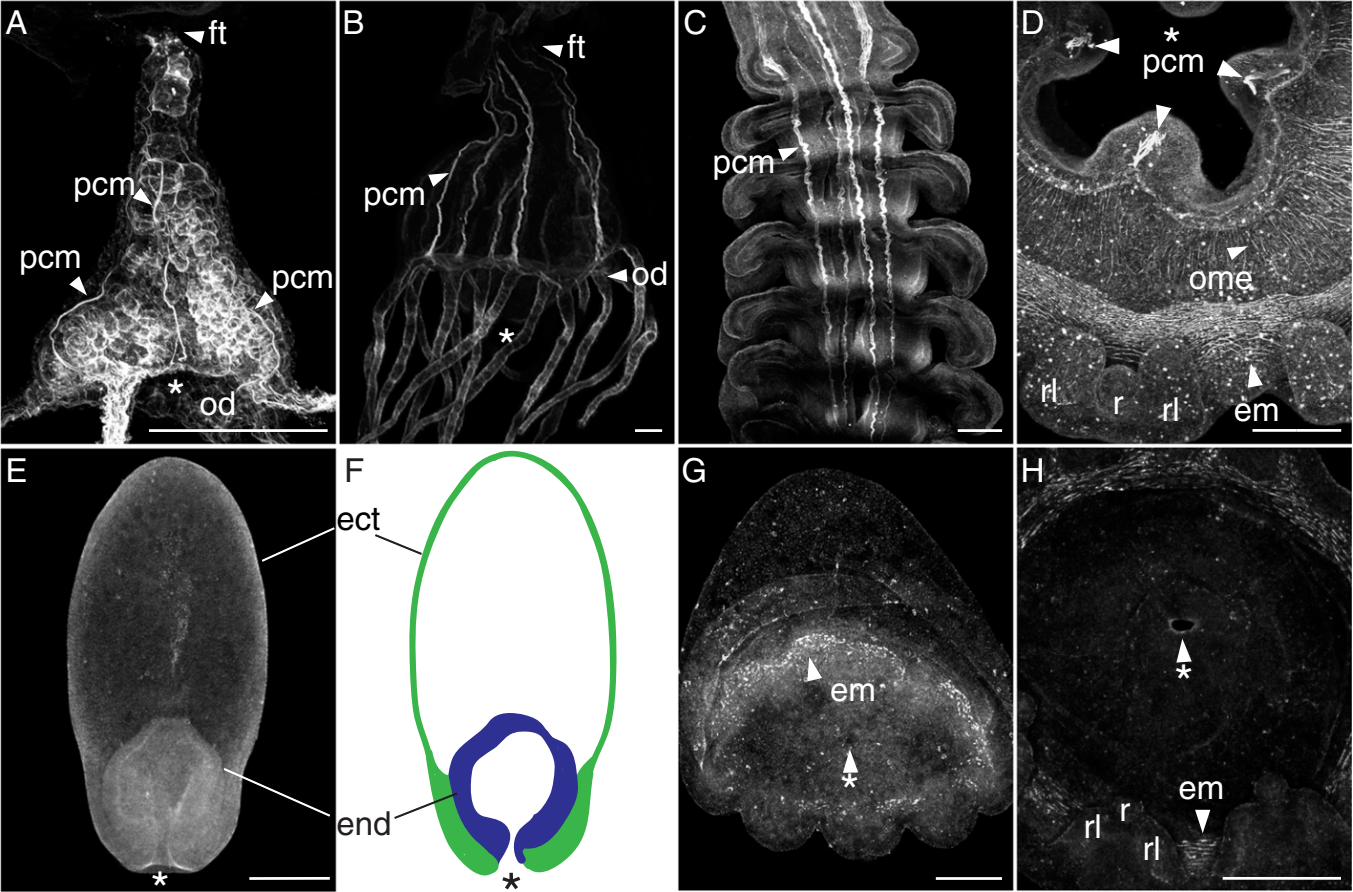
Cord muscle is present in polyps and strobilae of *C. quinquecirrha* but absent in *P. noctiluca*. Figures are oriented oral end down with lateral views unless otherwise noted. Muscle structures are stained with BODIPY Phallacidin **(A, B, C, E, G)** or alexa-fluor phalloidin **(D, H)**, and generated on a confocal microscope, except **(D)**, which was imaged on a multiphoton microscope. **(A)** A *Chrysaora quinquecirrha* polyp newly emerged from a podocyst, showing three visible polyp cord muscles (pcm) extending from the foot (ft) to the oral disk (od). **(B)** Large polyp showing six cord muscles (pcm) running from the foot (ft) to the oral disk (od). **(C)** Polyp cord muscles (pcm) in strobilae are centrally located and appear as stripes running along the oral-aboral axis. Aborally, these cord muscles are thick and well formed, growing thin orally as ephyrae mature and degrading completely after ephyrae detach. **(D)** Oral view of a developing ephyra cut from a strobila, showing three of the four polyp cord muscles (pcm) around the immature ephyra mouth, developing ephyra muscle (em) around the margin near the rhopalial lappets (rl) and rhopalia (r), and oral myoepithelia (ome) bundles radiating from the corners of the mouth. **(E)** *Pelagia noctiluca* at the four-prong stage showing no evidence of cord-like muscle on the oral-aboral axis. **(F)** Diagram of the same four-prong stage showing the endoderm (end, blue) and ectoderm (ect, green). **(G)** Oblique view of a *P. noctiluca* cone larva (compressed laterally) with small arm buds forming, and actin-rich bundles of developing ephyra muscle (em) around the circumference of the future mouth. No cord-like muscles are present. **(H)** An oral view of a more mature *P. noctiluca* showing no evidence of cord-like muscle around the mouth but with developing rhopalia. * = Mouth. All scale bars are 100 μm.

Animals were relaxed in isosmotic magnesium chloride for 5 to 10 min prior to fixation. Animals were fixed for 2 h in a 4% paraformaldehyde 0.2% glutaraldehyde solution with 0.2-μm filtered seawater. After fixation, animals were moved from fixative to seawater and then gradually transferred to 100% phosphate-buffered saline with 1% Triton X-100 (PBT). 50 μl of phallotoxin (manufacturer-recommended stock concentration) was evaporated and reconstituted with 1-mL PBT and added to samples (5 to 10 strobilae, 100 to 200 *P. noctiluca* larvae), which were stained for 2 h rocking in the dark. TRITC phalloidin (Sigma-Aldrich; catalog number: 77418) was used for most *P. noctiluca* samples, and BODIPY FL Phallacidin (Life Technology; catalog number: B607) for all *C. quinquecirrha* samples, and the *P. noctiluca* four-prong stage (a stage that superficially resembles an early polyp, see ‘Characterization of *Pelagia noctiluca* development’). These phallotoxins both stain filamentous actin, but one (BODIPY) can be used with clearing agents because it does not fade as rapidly in alcohol. Most *P. noctiluca* samples were imaged directly after staining with a Leica confocal microscope (TCS SP5) at Observatoire Océanologique de Villefranche-sur-mer. More opaque samples, including *C. quinquecirrha* polyp samples and *P. noctiluca* four-prong samples, were cleared before imaging. To clear, samples were first dehydrated to 100% isopropanol with a dilution series of 10%, 25%, 50%, 75%, 90%, and 100% (2×) water:isopropanol for 30 s each, and then washed two to three times with BABB (50% benzyl benzoate, 50% benzyl alcohol). For Figure 2D and Additional file 1: Figure S1A,B, a multiphoton microscope (Olympus FV1000-MPE) without clearing provided the best resolution of the fine oral myoepithelial bundles, because it did not image deep enough to pick up the background from autofluorescent endodermal cells. Remaining samples were imaged at Brown University with a Zeiss LSM 510 confocal microscope. All image processing was done with FIJI [18]. For some images, value histograms were adjusted in Photoshop to include the full value range.

### Cord muscle removal

To test if polyp cord muscle is necessary for development of ephyra muscle, we isolated developing ephyrae from four strobila stacks, for a total of 16 ephyra disks at a range of maturities. We then isolated sections of the margin from each disk, eliminating polyp cord muscle. Cord muscle occurs near the future mouth; by separating the oral region from the margin, we removed cord muscle from developing lappets. All ephyra segments were checked daily for signs of movement, and after four days all ephyra segments were pulsing. Ten segments were video/photo documented, fixed, stained and imaged for signs of muscle development.

## Results and discussion

### Characterization of cord muscle in polyps and strobilae of *Chrysaora quinquecirrha*

We first characterized polyp muscle morphologies in *C. quinquecirrha*, to confirm previous findings and validate our methods. Scyphozoan polyps have four to six well-defined ectodermally derived cord muscles that extend the length of the polyp body [19]. Polyps also possess muscle fibers in the oral disk and tentacles [20]. However, we chose to focus only on cord muscle, because this muscle group is the only polyp muscle type present in all developing ephyrae. The polyp cord muscles are clearly visible in *C. quinquecirrha* with our methods (Figure 2A-D). Each cord muscle attaches to the oral disk at a peristomal pit [19] and run the length of the body, terminating near the foot (Figure 2A-B). These cord muscles are present even in very small polyps (Figure 2A).

Polyp cord muscle is reported to persist in the strobila, running from the aboral apex of one developing ephyra, through the ephyra mesoglea, and out of the grooves of the developing ephyra mouth and into the aboral surface of the next ephyra [21]. We confirm this for *C. quinquecirrha* strobila (Figure 2C). Cord muscle grows thin in well-developed ephyrae, and the last remaining vestige of cord muscle disappears when an ephyra is liberated [21].

In contrast to polyp muscle morphology, ephyrae have two groups of striated muscle that persist into the adult medusa: a ring of ‘circular muscle’ running the circumference at the margin on the subumbrella, and ‘radial muscles’ that extends from the circular muscle towards the tips of the swimming lappets (Figure 1A,B). Ephyrae also have non-striated myoepithelial processes that run from the corners of the manubrium to the margin and are presumably associated with mouth movement. The developmental origin of ephyra muscles has not been previously described. They could arise *de novo* (consistent with compartmentalization) or be remodeled from polyp muscle.

### Polyp cord is not remodeled to form ephyra muscle in *Chrysaora quinquecirrha*

**Figure 3:**
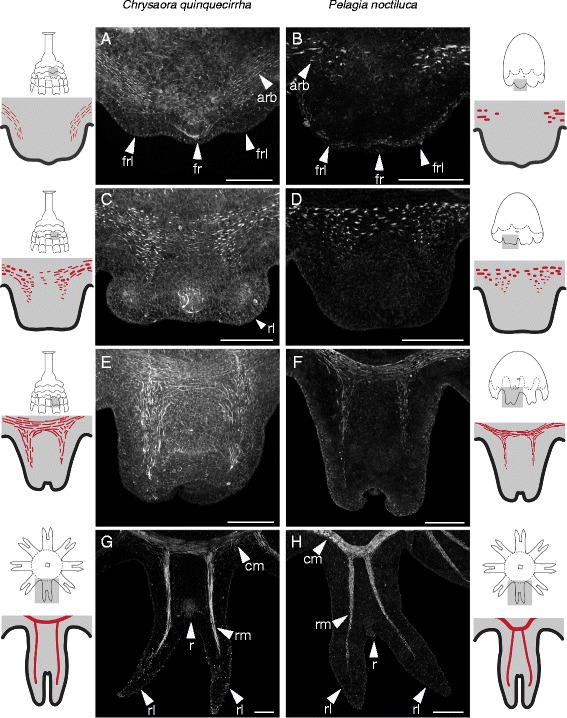
Ephyra muscle forms *de novo* in *P. noctiluca* and *C. quinquecirrha*, regardless of life cycle. **(A, B)** For both species, the first signs of ephyra muscle are actin-rich bundles (arb) found oral to the future rhopalia (fr) at the base of each future rhopalial lappet (frl). **(C, D)** Slightly later, actin-rich bundles are more numerous at the site of future radial and circular muscle. **(E, F)** As the ephyrae develop and rhopalial lappet curl orally (away from the viewer), actin-rich bundles appear elongate and striated. **(G, H)** At maturity, muscle extends around the ephyra as circular muscle (cm) and into the rhopalial lappets (rl) on either side of the rhopalia (r) as radial muscle (rm). Scale bar **(A-F)** 50 μm, **(G-H)** 100 μm.

Cord muscle within the strobila diminishes as *C. quinquecirrha* ephyrae mature, but it was not clear if this is due to senescence or to cord muscle remodeling in the oral ectoderm to form ephyra musculature. We examined ephyra formation to answer this question. In *C. quinquecirrha*, we examined the oral ectoderm of developing ephyrae at multiple stages and found no evidence that cord muscle contributes to ephyra muscle formation. When a developing ephyra is cut from the strobila and viewed orally, cord muscle is present between the corners of the future ephyra mouth (Figure 2C), while ephyra swimming muscle forms near the bell margin (Figures 2C and 3). We did, however, observe actin-rich bundles that run from the corners of the developing ephyra mouth to the margin, which we presume to be developing oral myoepithelial processes (Figure 2D). These oral myoepithelial processes are spatially separated from cord muscle, with cord muscle being nested between these processes. These processes also persist in mature ephyrae (Additional file 1: Figure S1). Polyp cord muscle appears consolidated through strobilation, with cord muscle actin diminishing as ephyrae mature and being completely lost in liberated ephyrae (Additional file 1: Figure S1).

To test the role of polyp cord muscle in ephyra muscle formation, we next isolated the margins of developing ephyrae at multiple developmental stages, effectively removing cord muscle and surrounding tissue in the process. All ephyra sections still produced lappets with pulsing movement and muscle (Additional file 2: Figure S2). Stained and imaged ephyra segments all possessed radial muscle and components of circular muscle. In one ephyra segment, we also observed possible oral myoepithelial processes (Additional file 2: Figure S2). These data present additional evidence that polyp cord muscle is not remodeled to produce ephyra circular or radial muscle, since its removal does not appear to inhibit muscle formation.

### Characterization of *Pelagia noctiluca* development

We next characterized development in the direct-developing *P. noctiluca* to look for evidence of a cryptic polyp and cord muscle (Additional file 3: Figure S3). Planulae in *P. noctiluca* have a unique morphology compared to planulae of other scyphozoans [22]. The endoderm remains consolidated at the oral end, and in late planulae, this endoderm is asymmetric with one large pouch and one small pouch flanking the archenteron/oral opening ([14], Additional file 3: Figure S3). As planulae develop, a transient morphology forms that superficially resembles a metamorphosing polyp, which we call the ‘four-prong stage’ (Figure 2E). At this stage, the *P. noctiluca* oral end is squared with four buds around the mouth (Additional file 3: Figure S3). This form is superficially similar to the squared morphology of metamorphosing moon jelly planulae (*Aurelia aurita*), with a square-shaped oral surface and four polyp tentacle buds [22]. In *P. noctiluca*, soon after the formation of the four-prong stage, the larva expands orally, developing into a form we termed a ‘cone larva’ (Additional file 3: Figure S3). Four additional buds develop between the original buds in the four-prong stage, for a total of eight. As development progresses, each of these buds develops into a pair of rhopalial lappets with a nested rhopalia. The cone larva then flattens along the oral aboral axis, and a recognizable ephyra morphology is formed. We suspected the four-prong stage may be a cryptic polyp and next looked for evidence of polyp muscle morphology in this and other stages.

### *Pelagia noctiluca* do not have polyp cord muscle

We examined stages from early planula to ephyra for evidence of cord muscle, focusing particularly on the four-prong stage. In *C. quinquecirrha* polyps of even smaller size, cord muscle is clearly visible running along the oral-aboral axis (Figure 2A). However, we found no evidence of cord muscle along the oral-aboral axis at any stage of *P. noctiluca* development, even during the four-prong stage (Figure 2E). In the four-prong stage, a mouth opening is clearly visible, connected to a hollow endodermal cavity that occupies the first quarter of the oral half (Figure 2E,F), with the aboral region of the four-prong stage being an extracellular matrix lined by ectoderm (Figure 2E,F). No actin-rich cells were seen between the ectoderm and endoderm or under the ectoderm in the aboral region. We next looked at the developing *P. noctiluca* mouth, corresponding to the region where polyp cord muscle attached to the oral disk. In *P. noctiluca*, we found no evidence of cord muscles around the mouth at any point in development (Figure 2G,H). Like in *C. quinquecirrha*, we did observe evidence of developing oral myoepithelial processes (Additional file 1: Figure S1), which appear to arise *de novo*. Thus, there is no evidence of polyp-like musculature in *P. noctiluca*.

### Ephyra swimming muscle arises *de novo* in both species

Even though *P. noctiluca* and *C. quinquecirrha* have very different life cycles, we found the development and morphology of medusa muscle to be quite similar. In both species, the morphogenesis of each swimming arm (rhopalium and associated rhopalial lappets) is first seen as a small bud on the rim of the oral surface (Figure 1). The first signs of muscle are actin-rich bundles in the subumbrellar ectoderm of these buds (Figure 3A,B). At this stage, the rhopalia are forming and visible, and actin-rich bundles are localized to the base of each future rhopalial lappet and offset to the side of future rhopalia, such that no actin-rich bundles could be seen orally of the rhopalia (Figure 3A,B). In *C. quinquecirrha* (Figure 3A), these actin-rich bundles appear narrower than in *P. noctiluca*. Slightly later, as the rhopalial lappets became more differentiated, actin-rich bundles grow more numerous and became visible orally to the rhopalia (Figure 3C,D). At this stage, a contiguous band of actin-rich bundles stretches around the site of future circular muscle. Actin-rich bundles are also visible at the site of future radial muscle, and their orientation at this stage is largely circular (Figure 3C,D). When the rhopalial lappets and statocyst are well differentiated and the lappets begin to curl orally, muscle striation is evident (Figure 3E,F). Actin-rich bundles are now oriented either radially or circularly, depending on their location in radial or circular muscle (Figure 3E,F). These actin-rich bundles are loosely connected, forming bands of muscle that extend around the circumference as circular muscle and into the developing lappets as radial muscle. At this stage, gentle pulsing is seen in *P. noctiluca* and possibly in *C. quinquecirrha*, though movement of whole strobilae made observing minute movements in early ephyrae difficult. Mature muscle is seen in liberated ephyrae in *C. quinquecirrha,* and in *P. noctiluca* ephyrae that have transitioned completely from the cone-shaped morphology to an oral-aboral flattened morphology (Figure 3G,H). At this stage, both circular and radials muscle are composed of long striated muscle fibers. Circular muscle stretches around the bell and radial muscle extends into the rhopalial lappets (Figure 3G,H).

To see if this type of muscle development is present in other *Chrysaora* species, we also examined ephyra development in *Chrysaora achlyos*, a second pelagiid species with a polyp (Additional file 4: Figure S4). This data set is limited, as we were not able to image cord muscle morphology in polyps or strobilae (due to a limited number of animals), but the presence and abundance of actin-rich bundles during ephyra development is broadly similar to both *P. noctiluca* and *C. quiqnuecirrha* (Additional file 4: Figure S4). In early ephyrae, actin-rich bundles are present at the base of developing rhopalial lappets; and in later ephyrae, the bundles grow more numerous in the regions of future circular and radial muscle, ultimately elongating to form functional circular and radial muscle groups. The broad similarity of ephyra muscle development in these three species suggests the process of ephyra muscle development is conserved in the Chrysaora clade (including *P. noctiluca*).

## Conclusions

### Implications for life cycle evolution

Our investigations of muscle morphology indicate that ephyra muscle arises *de novo* and in a similar way in *C. quinquecirrha* and *P. noctiluca*, despite radically different life cycles. Polyp muscle is not remodeled for ephyra circular or radial muscle formation in the complex life cycle of *C. quinquecirrha*, and we found no evidence of polyp-like muscle in *P. noctiluca*, a species with a simplified life cycle that lacks a benthic stage*.*

If polyp muscle is not remodeled to form ephyra musculature, one possibility is that ephyra muscle formation is compartmentalized [15]. Compartmentalization may have facilitated the evolutionary origin of a simplified life cycle in *P. noctiluca*, because the development of adult morphology is ‘decoupled’ from larval morphology [15]. Yet, compartmentalization in many animal life cycles is achieved with set-aside cells [15]. Some hydrozoans have multipotent cells, known as i-cells, and this cell type would be a good candidate for set-aside cells in Pelagiidae. However, no i-cells, stem-like cells, or progenitor cells have been identified in Scyphozoa [23]. How might developmental decoupling between polyps and medusae be achieved?

There are at least two alternatives. First, it is possible that as-yet unidentified multipotent or progenitor cells are present in scyphozoans, and these cells facilitate compartmentalization. Assays that label dividing cells, such as BrdU, in combination with markers for transcripts associated with multipotent cells (such as *vasa*, *piwi*, or *nanos*) will help clarify if scyphozoans have multipotent cells and their possible role in ephyra development. Second, Schmid et al. [24] reported transdifferentiation of hydrozoan muscle cells in culture, where mature muscle cells lost their myofibers, developed a crawling morphology to spread, and then re-developed muscle structures. Cellular transdifferentiation may be an important component of metamorphosis in Scyphozoa, where previously differentiated polyp cells transdifferentiate to become different cell types in ephyrae. Transdifferentiation could give rise to *de novo* ephyra muscle and represents a process that involves both remodeling of existing tissue, since differentiated cells are giving rise to new structures, as well as compartmentalization, since different developmental programs are being deployed during differentiation. Demonstrating transdifferentiation potential in Scyphozoa, using similar methods as Schmid et al. [24], would be a first step to testing this hypothesis.

Regardless of the developmental mechanism by which developmental decoupling of polyps and ephyrae is achieved, other observations of scyphozoans are consistent with our results that polyp morphology is not necessary for ephyra formation. Under certain environmental conditions, the planulae of the moon jellyfish *A. aurita* have been reported to metamorphose directly into ephyrae, seemingly bypassing the normal polyp stage [25,26]. This facultative direct development from a planula to ephyra may provide additional insights into how obligatory direct development evolved in *P. noctiluca*. Just as ephyrae can form from planulae in some instances, polyps can also form from ephyra-related structures. *Aurelia aurita* strobilae can revert to forming chains of polyps, rather than stacks of ephyrae, if exposed to environmental stress [26]. These observations suggest that different life cycle stages are capable of forming from a variety of tissue types at different times, even in species with canonical complex life cycles.

In this study, we only examine development of muscle, and our results that muscle is not remodeled may not translate to the development of other ephyra morphologies. Similarly, absence of polyp-like muscle in *P. noctiluca* larvae does not exclude the possibility that other polyp morphologies are recapitulated in *P. noctiluca* development. Examining other polyp features, such as the nerve net, will help shed greater light on ephyra metamorphosis and life cycle evolution. Our results do suggest that the retention of a transient polyp stage in *P. noctiluca* may not have been necessary for ephyra muscle formation. If polyp morphologies are indeed unnecessary to forming ephyrae, a simple shift in developmental timing may have sufficiently enabled the evolution of a direct life cycle in *P. noctiluca*.

## Additional files

Additional file 1:
**Helm-EvoDevo-Figure S1.** Diminishing cord muscle in developing *C. quinquecirrha* ephyrae and developing oral myoepithelial bundles in *C. quinquecirrha* and *P. noctiluca*. (A) Polyp cord muscle (pcm) is present in *C. quinquecirrha* young ephyrae with newly forming lappets, along with developing oral myoepithelial (ome) bundles. (B) At a later stage in *C. quinquecirrha* ephyra development, polyp cord muscle (pcm) persists near the mouth, and oral myoepithelial (ome) bundles become more numerous. (C) In late-stage ephyrae, oral myoepithelial (ome) bundles are well developed and spread from the corners of the manubrium to the margin; a close-up of the mouth (green box) (D) shows polyp cord muscle (pcm) is greatly diminished, with only small actin-rich bundles remaining. (E) A *P. noctiluca* cone larva with developing oral myoepithelial cells around the mouth, in the same region as *C. quinquecirrha* myoepithelial cells, and (F) a mature *P. noctiluca* ephyra, with oral myoepithelial bundles present and extending from the corners of the mouth to the circular musculature. All scale bars are 100 μm. Additional file 2:
**Helm-EvoDevo-Figure S2.** Experimental isolation of ephyra margin. Isolated ephyra margins from a range of developmental stages all developed into pulsing fragments with well-developed musculature. (A) A diagram of an isolated developing ephyra disk, showing developing lappets at the margin, a developing mouth (black), the location of polyp cord muscle (red), and the approximate location of the margin amputation site (black dotted line). (B) A diagram of a strobila, with grey developing ephyra disks representing the maturity of each of the three pictured animals at the time of margin amputation, with the approximate cut site of the amputated margin illustrated with the black dotted line. (C) Mature ephyrae margins after four days of development, with the cut site having close to form radial-like ephyrae. (D) The same segments stained with BODIPY Phallacidin, showing the location of radial muscle (rm), circular muscle (cm), rhopalial lappets (rl), rhopalia (r), and in one instance (middle right panel) oral myoepithelial (ome) processes and a mouth rudiment. All scale bars are 100 μm. Additional file 3:
**Helm-EvoDevo-Figure S3.** Early development of *Pelagia noctiluca. P. noctiluca* development begins when large eggs, which come in a variety of colors, are fertilized externally or internally and spawned roughly 2 h after first light exposure. (A) The first cleavage is unipolar (cf = cleavage furrow), and subsequent cleavages are equal though not ordered. (B) Blastulae are highly variable in shape and cleavage patterning. (C) Late blastulae have a characteristic ‘prawn chip’ (pc) morphology. (D) Some embryos have a centralized area of pigmentation (p) in each blastomere, which may be associated with nuclei. (E) Some early embryos also show lateral dark pigmentation (p), as in this four-cell embryo (top) and later gastrula stage (bottom). (F) Planulae possess a unique asymmetric morphology, (F-i) with a large endodermal cavity. (F-ii) Three different planulae stained with BODIPY Phallacidin and viewed with confocal microscopy, showing at least two endodermal pockets (pt) of variable sizes in each planula. (G-i) In some late stage planulae, one side of the oral disk protrudes asymmetrically, (G-ii) and all late-stage planulae square at the oral end as developing arm buds (bd) form. This stage superficially resembles the metamorphosing planula of some scyphozoans, and we refer to this stage as the ‘four-prong stage’, with each prong being fated to form a swimming arm (pair of rhopalial lappets and rhopalium). Four-prong larvae transition to a (H) cone-like morphology as the oral end swells and four secondary buds (bd) grow between the primary buds. (I) In later stages, a rhopalium rudiment, destined to form a future rhopalium (fr), is present at the end of each swimming arm. The timing of these stages is variable depending on clutch and culture density, but at 18°C follows roughly: first cleavage at 6.5-h post fertilization (hpf); ~128 cell stage at 25 hpf; swimming blastula at 48 hpf; planula stage at 55 hpf; four-prong stage at 60 hpf; early cone larva at 68 hpf; rhopalia primordia visible at 81 hpf; ephyra at 104 hpf. White asterisk = blastopore/mouth. All scale bars are 100 μm. Additional file 4:
**Helm-EvoDevo-Figure S4.** Development of *Chrysaora achlyos* medusa muscle. The process of muscle development in *C. achlyos* is broadly similar to that of *P. noctiluca* and *C. quinquecirrha*. (A) actin-rich bundles (arb) form at the base of the developing future rhopalial lappets (frl). (B) Actin-rich bundles become more numerous as the lappets mature and (C) eventually elongate to form clear tracks of radial and circular muscle, though striation was not found at this stage in this species. (D) These actin-rich bundles elongate and striate to form mature radial muscle (rm) and circular muscle (cm) in liberated ephyrae. r = rhopalia, rl = rhopalial lappet. These specimens were imaged using the same methods as *C. quinquecirrha*. All scale bars are 100 μm.

## Abbreviations

*: mouth
arb: actin-rich bundles
bd: bud
cf: cleavage furrow
cm: circular muscle
ect: ectoderm
em: ephyra muscle
end: endoderm
fr: future rhopalia
frl: future rhopalial lappet
ft: foot
od: oral disk
ome: oral myoepithelia
p: pigment
pc: prawn chip
pcm: polyp cord muscle
pt: pocket
r: rhopalia
rl: rhopalial lappets
rm: radial muscle

## References

[1] Bahir MM, Meegaskumbura M (2005). Reproduction and terrestrial direct development in Sri Lankan shrub frogs (Ranidae: Rhacophorinae: Philautus). Raffles Bull Zool.

[2] Smith MS, Zigler KS, Raff RA (2007). Evolution of direct-developing larvae: selection vs loss. BioEssays.

[3] Kulkarni SS, Singamsetty S, Buchholz DR (2010). Corticotropin-releasing factor regulates the development in the direct developing frog, *Eleutherodactylus coqui*. Gen Comp Endocrinol.

[4] Kerney RR, Blackburn DC, Müller H, Hanken J (2011). Do larval traits re-evolve? Evidence from the embryogenesis of a direct-developing salamander*, Plethodon cinereus*. Evolution.

[5] Kerney R, Gross JB, Hanken J (2010). Early cranial patterning in the direct‐developing frog *Eleutherodactylus coqui* revealed through gene expression. Evol Dev.

[6] Morandini AC, Marques AC (2010). Revision of the genus *Chrysaora* Peron and Lesueur, 1810 (Cnidaria: Scyphozoa). Zootaxa.

[7] Piraino S, Aglieri G, Martell L, Mazzoldi C, Melli V, Milisenda G (2014). Pelagia benovici sp. nov. (Cnidaria, Scyphozoa): a new jellyfish in the Mediterranean Sea. Zootaxa.

[8] Gershwin LA, Zeidler W (2008). Two new jellyfishes (Cnidaria: Scyphozoa) from tropical Australian waters. Zootaxa.

[9] Lynam CP, Gibbons MJ, Axelsen BE, Sparks C (2006). Jellyfish overtake fish in a heavily fished ecosystem. Curr Biol.

[10] Purcell JE, Uye S, Lo WT (2007). Anthropogenic causes of jellyfish blooms and their direct consequences for humans: a review. Mar Ecol Prog Ser.

[11] Collins A (2002). Phylogeny of Medusozoa and the evolution of cnidarian life cycles. J Evol Biol.

[12] Bayha KM, Dawson MN, Collins AG, Barbeitos MS, Haddock SHD (2010). Evolutionary relationships among scyphozoan jellyfish families based on complete taxon sampling and phylogenetic analyses of 18S and 28S ribosomal DNA. Integr Comp Biol.

[13] Metchnikoff E (1886). Embryologische Studien an Medusen.

[14] Goette A (1893). Vergleichende Entwicklungsgeschichte von Pelagia noctiluca Pér. Z Wiss Zool.

[15] Moran NA (1994). Adaptation and constraint in the complex life cycles of animals. Annu Rev Ecol Syst.

[16] Levine RB, Truman JW (1982). Metamorphosis of the insect nervous system: changes in morphology and synaptic interactions of identified neurons. Nature.

[17] Kuniyoshi H, Okumura I, Kuroda R, Tsujita N, Arakawa K, Shoji J, et al. Indomethacin induction of metamorphosis from the asexual stage to sexual stage in the moon jellyfish Aurelia aurita. Bioscience. 2012;76:1397–400. 10.1271/bbb.120076 22785488

[18] Schindelin J, Arganda-Carreras I, Frise E, et al. Fiji: an open-source platform for biological-image analysis. Nature methods. 2012;9:676–682. 10.1038/nmeth.2019 PMC3855844 22743772

[19] Chapman DM. Evolution of the Scyphistoma. Symp Zool Soc Lond. 1966;16:51–75.

[20] Chia FS, Amerongen HM, Peteya DJ. Ultrastructure of the neuromuscular system of the polyp of Aurelia aurita L., 1758 (Cnidaria, Scyphozoa). J Morphol. 1984;180:69–79. 10.1002/jmor.1051800108 30041506

[21] Russell FS. The medusae of the British Isles: pelagic Scyphozoa with a supplement to the first volume on hydromedusae. London: Cambridge University Press; 1970.

[22] Yuan D, Nakanishi N, Jacobs D, Hartenstein V. Embryonic development and metamorphosis of the scyphozoan Aurelia. Development Genes and Evolution. 2008;218:525–539. 10.1007/s00427-008-0254-8 18850238

[23] Gold DA, Jacobs DK. Stem cell dynamics in Cnidaria: are there unifying principles? Dev Genes Evol. 2012;223:53–66. 10.1007/s00427-012-0429-1 PMC7211294 23179637

[24] Schmid V, Baader C, Bucciarelli A, ReberMüller S. Mechanochemical interactions between striated muscle cells of jellyfish and grafted extracellular matrix can induce and inhibit DNA replication and transdifferentiation in vitro. Dev Biol.1992;155:483–96. 10.1006/dbio.1993.1046 8432401

[25] Haeckel EHPA. Metagenesis und Hypogenesis von Aurelia Aurita. Jena: G. Fischer; 1881.

[26] Kakinuma Y. An experimental study of the life cycle and organ differentiation of Aurelia aurita Lamarck. Bull Mar Biol Stat Asamushi. 1975;15:101–16.

