# Supplementary figures and images for: Comparative muscle development of scyphozoan jellyfish with simple and complex life cycles
Source: EvoDevo. 2015 Apr 17;6:11. doi: 10.1186/s13227-015-0005-7 (PMC4415277; doi:10.1186/s13227-015-0005-7)

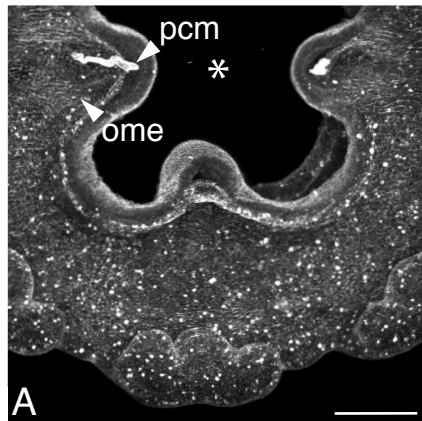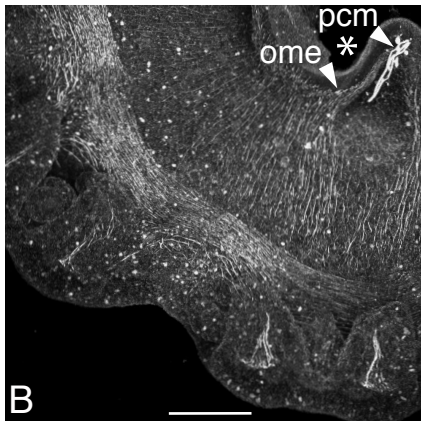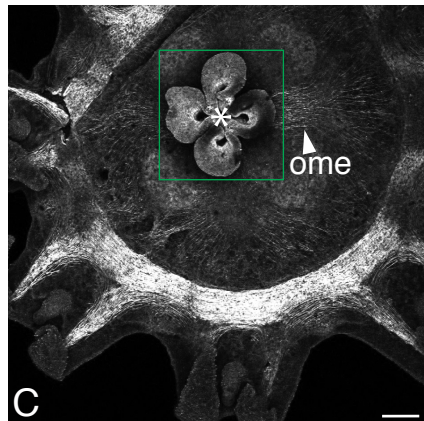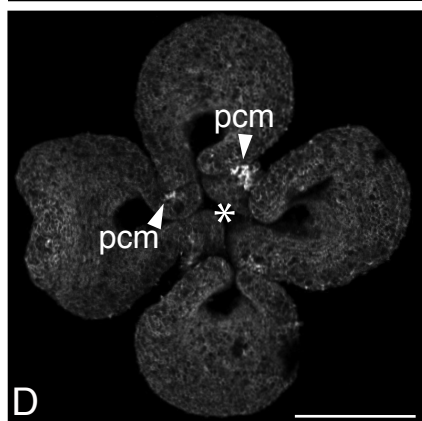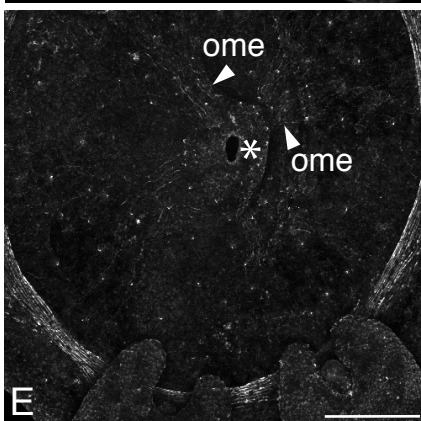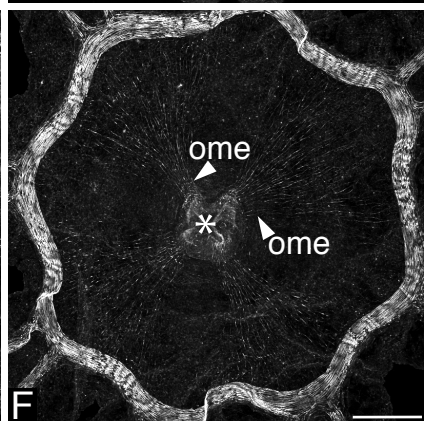

Supplement: Additional file 1: — Helm-EvoDevo-Figure S1. Diminishing cord muscle in developing C. quinquecirrha ephyrae and developing oral myoepithelial bundles in C. quinquecirrha and P. noctiluca. (A) Polyp cord muscle (pcm) is present in C. quinquecirrha young ephyrae with newly forming lappets, along with developing oral myoepithelial (ome) bundles. (B) At a later stage in C. quinquecirrha ephyra development, polyp cord muscle (pcm) persists near the mouth, and oral myoepithelial (ome) bundles become more numerous. (C) In late-stage ephyrae, oral myoepithelial (ome) bundles are well developed and spread from the corners of the manubrium to the margin; a close-up of the mouth (green box) (D) shows polyp cord muscle (pcm) is greatly diminished, with only small actin-rich bundles remaining. (E) A P. noctiluca cone larva with developing oral myoepithelial cells around the mouth, in the same region as C. quinquecirrha myoepithelial cells, and (F) a mature P. noctiluca ephyra, with oral myoepithelial bundles present and extending from the corners of the mouth to the circular musculature. All scale bars are 100 μm. [file 13227_2015_5_MOESM1_ESM.pdf]

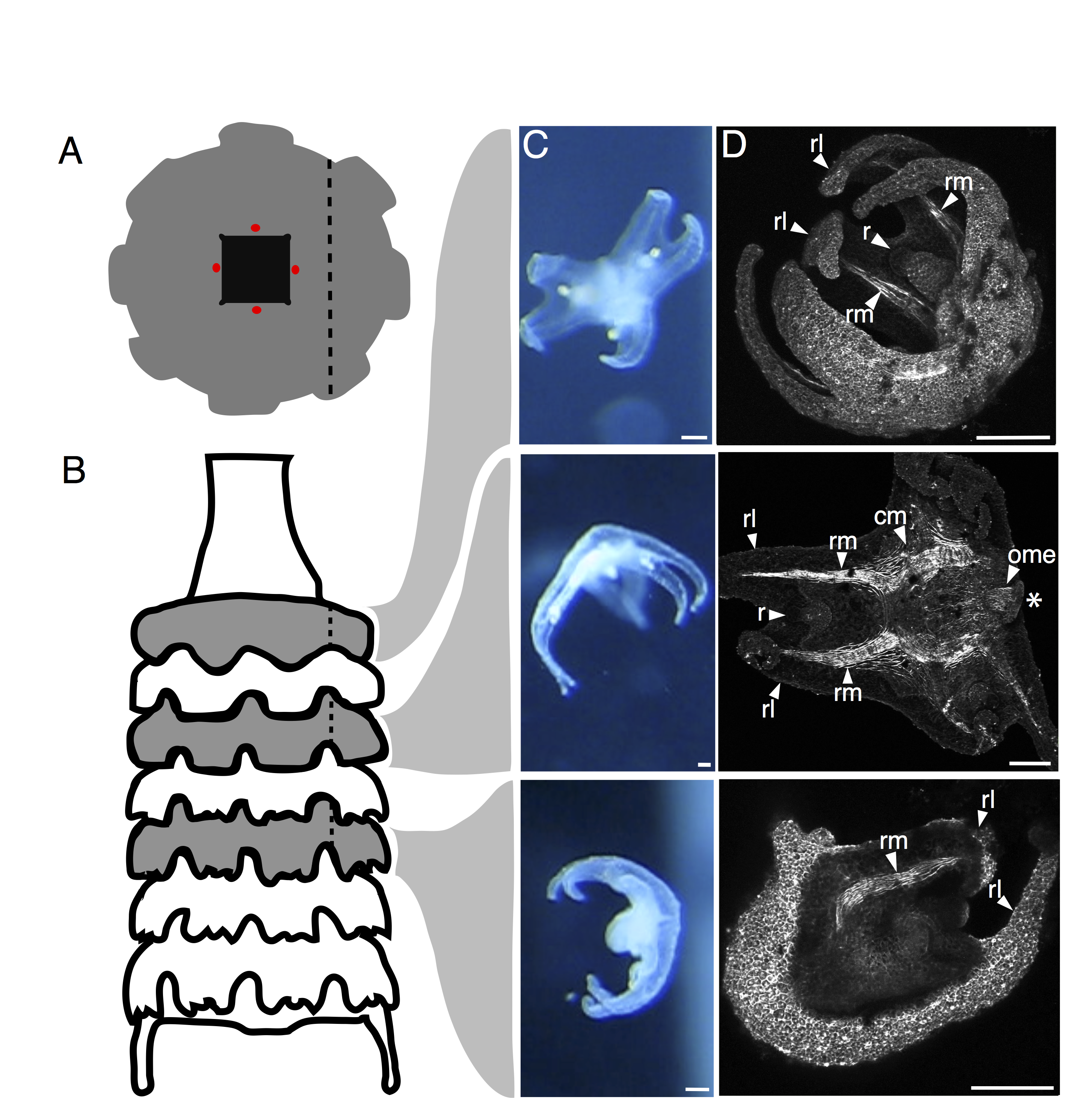

Supplement: Additional file 2: — Helm-EvoDevo-Figure S2. Experimental isolation of ephyra margin. Isolated ephyra margins from a range of developmental stages all developed into pulsing fragments with well-developed musculature. (A) A diagram of an isolated developing ephyra disk, showing developing lappets at the margin, a developing mouth (black), the location of polyp cord muscle (red), and the approximate location of the margin amputation site (black dotted line). (B) A diagram of a strobila, with grey developing ephyra disks representing the maturity of each of the three pictured animals at the time of margin amputation, with the approximate cut site of the amputated margin illustrated with the black dotted line. (C) Mature ephyrae margins after four days of development, with the cut site having close to form radial-like ephyrae. (D) The same segments stained with BODIPY Phallacidin, showing the location of radial muscle (rm), circular muscle (cm), rhopalial lappets (rl), rhopalia (r), and in one instance (middle right panel) oral myoepithelial (ome) processes and a mouth rudiment. All scale bars are 100 μm. [file 13227_2015_5_MOESM2_ESM.png]

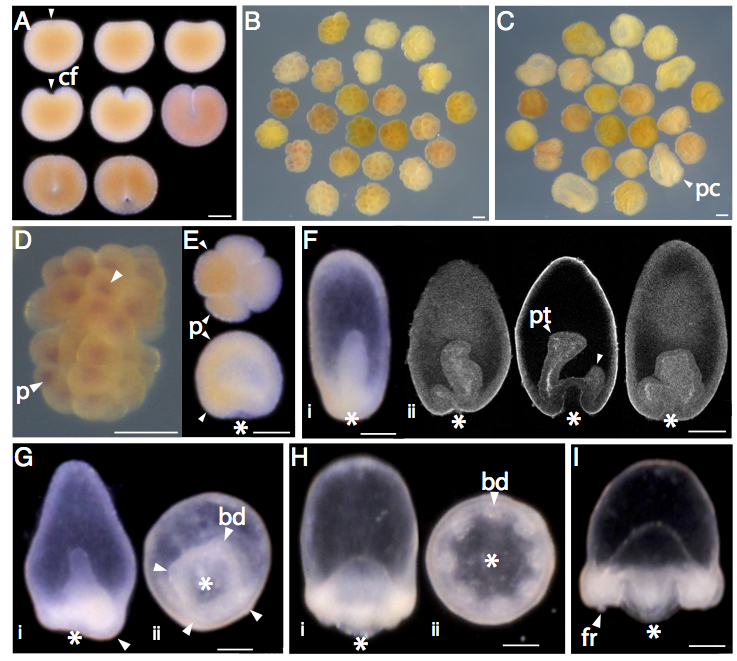

Supplement: Additional file 3: — Helm-EvoDevo-Figure S3. Early development of Pelagia noctiluca. P. noctiluca development begins when large eggs, which come in a variety of colors, are fertilized externally or internally and spawned roughly 2 h after first light exposure. (A) The first cleavage is unipolar (cf = cleavage furrow), and subsequent cleavages are equal though not ordered. (B) Blastulae are highly variable in shape and cleavage patterning. (C) Late blastulae have a characteristic ‘prawn chip’ (pc) morphology. (D) Some embryos have a centralized area of pigmentation (p) in each blastomere, which may be associated with nuclei. (E) Some early embryos also show lateral dark pigmentation (p), as in this four-cell embryo (top) and later gastrula stage (bottom). (F) Planulae possess a unique asymmetric morphology, (F-i) with a large endodermal cavity. (F-ii) Three different planulae stained with BODIPY Phallacidin and viewed with confocal microscopy, showing at least two endodermal pockets (pt) of variable sizes in each planula. (G-i) In some late stage planulae, one side of the oral disk protrudes asymmetrically, (G-ii) and all late-stage planulae square at the oral end as developing arm buds (bd) form. This stage superficially resembles the metamorphosing planula of some scyphozoans, and we refer to this stage as the ‘four-prong stage’, with each prong being fated to form a swimming arm (pair of rhopalial lappets and rhopalium). Four-prong larvae transition to a (H) cone-like morphology as the oral end swells and four secondary buds (bd) grow between the primary buds. (I) In later stages, a rhopalium rudiment, destined to form a future rhopalium (fr), is present at the end of each swimming arm. The timing of these stages is variable depending on clutch and culture density, but at 18°C follows roughly: first cleavage at 6.5-h post fertilization (hpf); ~128 cell stage at 25 hpf; swimming blastula at 48 hpf; planula stage at 55 hpf; four-prong stage at 60 hpf; early cone larva at 68 hpf; rhopali [file 13227_2015_5_MOESM3_ESM.png]

*Chrysaora achlyos*

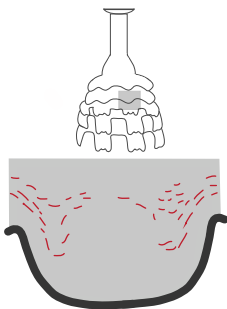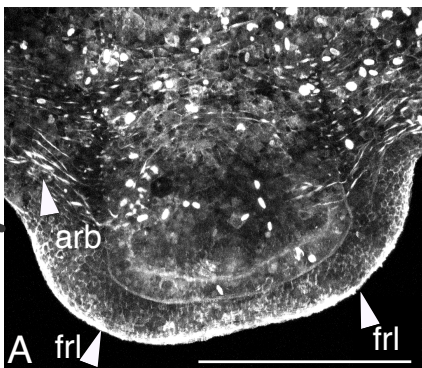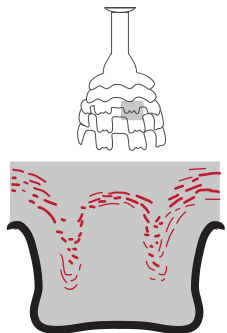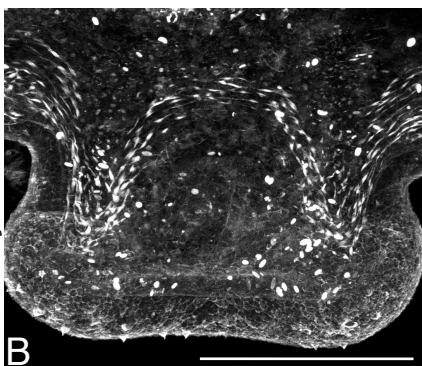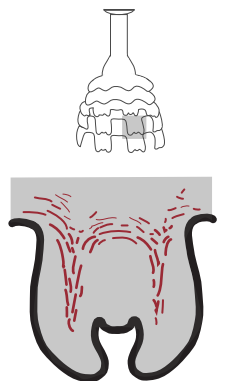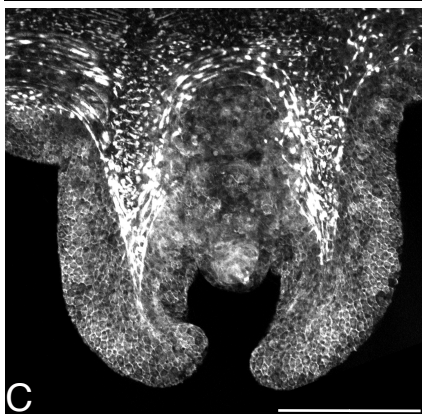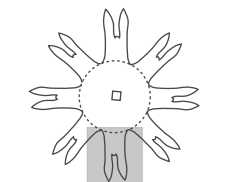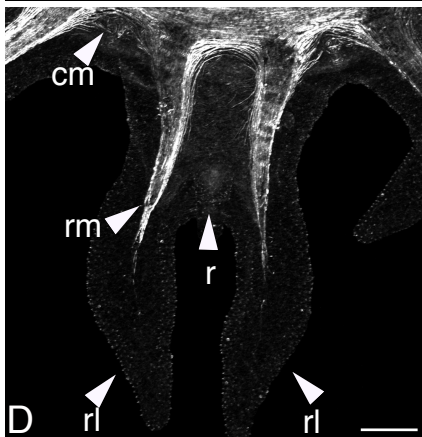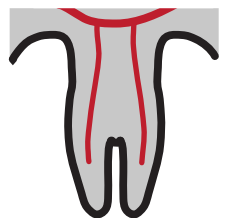

Supplement: Additional file 4: — Helm-EvoDevo-Figure S4. Development of Chrysaora achlyos medusa muscle. The process of muscle development in C. achlyos is broadly similar to that of P. noctiluca and C. quinquecirrha. (A) actin-rich bundles (arb) form at the base of the developing future rhopalial lappets (frl). (B) Actin-rich bundles become more numerous as the lappets mature and (C) eventually elongate to form clear tracks of radial and circular muscle, though striation was not found at this stage in this species. (D) These actin-rich bundles elongate and striate to form mature radial muscle (rm) and circular muscle (cm) in liberated ephyrae. r = rhopalia, rl = rhopalial lappet. These specimens were imaged using the same methods as C. quinquecirrha. All scale bars are 100 μm. [file 13227_2015_5_MOESM4_ESM.pdf]
